# The Predictive Value of Jump Height in Athletic Performance of Youth and Senior Soccer Players

**DOI:** 10.3390/sports14020058

**Published:** 2026-02-04

**Authors:** João G. Saldanha, Francisco Santos, Andreas Ihle, Rui Mâncio, Honorato Sousa, Hugo Sarmento, Élvio R. Gouveia

**Affiliations:** 1 Department of Physical Education and Sport, University of Madeira, 9020-105 Funchal, Portugal; joao.saldanha@staff.uma.pt (J.G.S.); francisco.j.santos@staff.uma.pt (F.S.); rui.caires@staff.uma.pt (R.M.); honorato.sousa@staff.uma.pt (H.S.); 2 Interactive Technologies Institute, LARSYS, 9020-105 Funchal, Portugal; 3 Swiss Center of Expertise in Life Course Research LIVES, 1227 Carouge, Switzerland; andreas.ihle@unige.ch; 4 Department of Psychology, University of Geneva, 1227 Carouge, Switzerland; 5 Center for the Interdisciplinary Study of Gerontology and Vulnerability, University of Geneva, 1227 Carouge, Switzerland; 6 Universidade de Coimbra, CIPER, Faculdade de Ciências do Desporto e Educação Física, 3004-531 Coimbra, Portugal; hg.sarmento@gmail.com; 7 CIPER, Faculdade de Motricidade Humana, Universidade de Lisboa, Cruz-Quebrada-Dafundo, 1649-004 Lisbon, Portugal

**Keywords:** countermovement jump, jump height, neuromuscular power, physical capacities, performance, football

## Abstract

Jump height (JH) is widely used as an indicator of athletic performance. This study aimed to (1) evaluate the relative importance and predictive value of JH for neuromuscular performance across key physical metrics and (2) describe the neuromuscular profile of soccer players from different age groups, positions, and competitive levels. Senior (SG) and youth (YG) players were evaluated after the off season for neuromuscular power, strength, change of direction, speed, repeated sprint ability, and aerobic endurance. SG outperformed YG in most measures, especially JH, abduction strength, and Peak Power (RAST PP). Notably, YG exhibited higher maximal oxygen uptake (VO2max) and lower fatigue index (RAST FI), highlighting their robust aerobic capacity and greater ability to sustain repeated efforts. These results reinforce established developmental patterns, with aerobic endurance more pronounced in youth and anaerobic power in seniors. In seniors, JH correlated moderately with sprint and anaerobic power, while its associations in youth were weaker and linked to endurance. Positional analysis suggested overall higher JH in SG. JH emerged as a practical predictor for physical performance monitoring in seniors and a useful benchmark for athletic potential identification. Findings support targeted training and monitoring based on age-specific profiles. This study enhances applied sports science, underscoring the need for tailored approaches in player development and evaluation.

## 1. Introduction

Soccer has been described as an acyclic and intermittent sport involving repeated high- to maximum-intensity actions (e.g., jumping, passing, shooting, tackling, changes of direction, and sprinting) interspersed with lower-intensity activities [1,2,3]. While these explosive actions represent a relatively small proportion of the total distance covered, they play a fundamental role, as many of them are often decisive for individual and collective success [2]. The physical demands of soccer have also increased in recent years, as match analyses report 30–80% increases in high-intensity running and sprint distances [4], with players in top European leagues typically covering 9 to 14 km per match, including approximately 900 m at high speed (>19.8 km·h^−1^), 300 m at sprint speed (>25.2 km·h^−1^), and performing more than 700 changes of direction [5,6,7,8,9]. These demands vary according to factors such as team and opposition quality, match outcome, tactical style, competitive level, playing position, and age [9,10,11]. Several studies [12,13,14,15,16] have shown that fitness levels improve from youth to adulthood, with elite players presenting higher strength, lower-body explosiveness, aerobic and anaerobic power, balance, and flexibility [17,18,19]. In addition to traditional physical fitness parameters, functional movement quality is an important factor in youth football performance, as Alexe et al. (2024) [3] reported that specific movement patterns moderately relate to speed, agility, and jump height (JH) in elite female youth players, indicating that movement efficiency may complement physical fitness assessments in athlete development.

The transition from youth to senior competition requires athletes to cope with increased physical, technical, tactical, and psychosocial demands [20,21]. Furthermore, biological maturation significantly influences physical characteristics in youth soccer players [22]. Research has shown that early-maturing males typically exhibit greater stature, muscle mass, and somatotype traits that favour speed and power. These maturity-related differences can impact talent identification and training strategies, emphasising the importance of considering maturation status when assessing and developing neuromuscular qualities such as JH [22]. Therefore, the development of well-rounded physical attributes, particularly strength and power, is essential for maximising performance and supporting a successful professional career.

Hence, physical fitness assessments are crucial for monitoring these attributes, as they (1) enable precise evaluation of fitness levels, (2) facilitate comparison with normative data, (3) help identify individual strengths and weaknesses, and (4) guide individualised training interventions [23,24]. Among athletes’ physical and functional assessments, the vertical jump test is extensively applied to analyse lower limb neuromuscular power [25,26,27]. While force platforms are the gold standard for measuring vertical jumps, portable alternatives such as infrared systems and contact mats provide cost-effective options suitable for in-field assessment [25]. The countermovement jump (CMJ), with JH as a fundamental parameter, is a common and practical test for evaluating athletic performance [2,28,29].

Additionally, JH is widely recognised as a key indicator of athletic potential, particularly in soccer, where it plays a relevant role in youth player selection for national teams [2,30]. The CMJ is also one of the most used jump tests for assessing neuromuscular readiness and fatigue status [31]. A recent systematic review highlights the CMJ, performed with hands fixed at the waist, as one of the most prevalent protocols in both research and practice, consistently reporting JH as the main metric [2]. Furthermore, the lower limbs’ stretch-shortening cycle (SSC) involves an eccentric muscle lengthening followed by a rapid concentric contraction. This neuromuscular function, commonly found in cyclical movements like running, enhances energy efficiency by reducing metabolic cost. The SSC enhances JH and force production through mechanisms such as increased residual force, stretch reflex activation, and passive tendon elasticity [32,33].

While physical performance supports many effective aspects of gameplay, it is important to note that it alone cannot fully capture the complexity of match performance. This study focuses on neuromuscular capacity through JH as a predictor of physical attributes and potential, providing actionable information for player development and training optimisation, which is distinct from tactical–technical proficiency or direct measures of on-field competitive success. Within this framework, JH is examined in relation to key physical performance metrics, including strength, change of direction, speed, repeated sprint ability, and aerobic endurance, supplemented by between-group and position-specific comparisons.

The thorough evaluation and monitoring of physical and physiological characteristics have led to the increasing use of diverse measurement technologies and devices, generating extensive data. As individualised, evidence-based monitoring becomes more prominent in elite football, understanding the relevance of each metric throughout different developmental stages is essential to foster more precise and effective training strategies.

Thus, based on prior experience with these athletes, we expected senior players to demonstrate superior physical performance across most metrics and hypothesised that JH would serve as a stronger predictor of performance in seniors compared with juniors. However, the relative contribution of JH across different age groups, playing positions, and the magnitude of performance differences remain to be elucidated. Our goal is to quantify the influence of JH as a predictor in each group and explore its significance within positional contexts, thereby providing comprehensive insights into neuromuscular profiles that better inform player monitoring and training decisions, taking into account sport specificity, competitive level, and positional demands.

## 2. Materials and Methods

### 2.1. Ethics

This study was carried out as part of the Marítimo Training Lab Project. The current project was conducted in accordance with the Declaration of Helsinki and approved by the Ethical Committee of the University of Lisbon, Faculty of Human Kinetics (CEIFMH, No. 34/2021). Informed written consent was obtained from all participants and young people’s legal guardians. To ensure confidentiality, data were anonymised by assigning a unique code to each player.

### 2.2. Sample

The sample comprised 80 male footballers, 36 of whom belonged to two professional senior teams (A and B) from the same club competing in the Portuguese 2nd and 4th divisions, respectively, and the remaining 44 individuals were from an under-19 team competing in the 1st National Division of their respective category. The subjects were categorised into two groups: the Senior Group (SG) and the Youth Group (YG). Players were also distributed by position (Goalkeeper (GK); Defender (DF); Midfielder (MF); and Forward (FW)), as shown in Table 1. The inclusion criteria consisted of all the players from all positions in different teams who correctly completed the execution of the applied tests. On the other hand, the exclusion criteria included players who were injured or had to be taken out of the tests due to contraindication orders from the medical department. Additional factors such as sleep quality, dietary habits, caffeine intake, and recent minor injuries were not systematically controlled or recorded. Although these factors were not fully controlled, all assessments were standardised (conducted in the morning under similar conditions during the initial pre-season phase), representing common constraints in applied elite sport settings where comprehensive control is often impractical.

### 2.3. Experimental Procedures

The assessments took place during the pre-season phase (2023/2024 and 2024/2025), on the first two days of the resumption of each group’s activities (SG and YG) after the off season.

Firstly, the research team measured and recorded anthropometric and demographic data (height, mass, birth date, position, dominant leg). Participants then performed a series of tests to analyse physical capacities, including vertical jumps, maximum hip adduction and abduction strength, maximum speed, change of direction, repeated sprinting ability and aerobic endurance. Prior to the repetitive tests, joint and muscle activation lasting approximately 10 min was conducted. The power, strength, and anaerobic tests, comprising CMJ, hip strength, maximum speed, change of direction, and repeated sprinting ability, were completed on a single day, while the aerobic endurance test was conducted on a separate day to minimise fatigue and allow sufficient recovery. Within the same day, sufficient rest was always ensured between tests, with a minimum interval of 2 to 3 min, as a large number of teammates meant that each player inevitably had to wait a few minutes for their turn. This sequencing follows common field-testing protocols in soccer, with similar adequate recovery periods between tests [34]. The order of testing was vertical jumps and hip strength (performed in a laboratory setting), followed by field tests in the sequence of maximum speed, change of direction, and repeated sprinting ability. All testing sessions took place in the morning under mild environmental conditions, and the field tests were conducted on dry grass surface to ensure consistency and control of external factors. Table 2 presents the total number of cases analysed per test variable.

#### 2.3.1. Vertical Jumps

The CMJ test was used to evaluate the vertical jumping capacity. Due to time restrictions, the protocol applied was the same as that outlined in Gouveia et al. (2023) [35]. Participants performed four data collection trials with 30 s of rest. The data were collected using the Optojump Next Version 1.13.24.0 (Microgate, Bolzano, Italy) system of analysis and measurement. Subjects positioned themselves between the parallel transmitting and receiving bars, placed on the floor, and aligned facing each other to create an optical measurement field of infrared LEDs and photocells. Before testing, the system was initialised via the Optojump software, with correct bar alignment and communication verified. Subjects were encouraged to jump to maximum height during testing. Prior to the protocol explanation, each participant was allowed three experimental trials, which served exclusively as familiarisation attempts to ensure correct execution. All familiarisation and recorded jumps were performed within the same session, with adequate rest between trials to allow for repeated maximal efforts without undue fatigue. In this test, players began standing, with feet placed hip-width to shoulder-width apart. Then, they dropped into nearly 90° of knee flexion from this position, followed by a maximal-effort vertical jump, with hands remaining on the hips for the entire movement. The trial was repeated if excessive knee flexion was observed or the hands were removed from the hips at any point. After completing each jump, participants reset to the starting position [35]. JH was automatically calculated from flight time using the standard equation implemented in the Optojump software. Previous research has demonstrated that vertical jump assessments, such as the CMJ, are recognised for their practicality and robust measurement consistency in athletic populations [36,37,38]. The CMJ specifically requires minimal familiarisation, is rapid to administer, and induces little fatigue, making it suitable for routine monitoring [36]. This type of jump consistently demonstrates excellent test–retest reliability, with intraclass correlation coefficients (ICC) regularly exceeding 0.90 and coefficients of variation (CV) typically ranging from 1.1% to <8.5% [36,37,38,39], confirming a high degree of reproducibility for these measures in sports settings. The performance metric taken into consideration for analysis was the highest value for JH. This approach reflects this study’s aim of examining associations between peak physical performance values and is consistent with established CMJ protocols utilising the best trial for maximal neuromuscular assessment [40,41,42].

#### 2.3.2. Maximum Hip Adduction and Abduction Strength

The maximum isometric strength of the hip adduction and abduction was estimated through the adductor and abductor squeeze test, using a portable dynamometer (Smart Groin Trainer, Neuro Excellence, Braga, Portugal). All tests were conducted by the same trained assessor, experienced in performing hip strength assessments in athletes. The assessment protocol was as follows [43,44,45]: (1) Subject lay on a mat on the floor in a supine position with their hips flexed 45° and their knees flexed 90°. (2) The examiner placed the dynamometer between the participant’s knees, specifically in the area of the medial femoral condyles. (3) Whenever it is a first-time assessment, the subject is given two attempts to familiarise with the test. (4) With the arms crossed in the chest area, the individual performed two 7 s repetitions, as the first quantified the force exerted by the left adductor and the second by the right adductor. (5) Once the two adduction repetitions were completed, the subject repeated the same procedure for abduction. (6) Passive recovery time between repetitions was 30 s and 1 min in between the adduction and abduction tests. For the assessment of hip adduction and abduction strength, no intra-rater reliability checks were performed in this sample. However, the literature on both football players and runners has established good to excellent intra-rater reliability when using handheld dynamometers. These studies report ICC values ranging from 0.76 to 0.98, supporting the validity and reproducibility of these strength protocols in adult male athletes [46,47]. The maximum force values obtained for both legs in adduction and abduction were subsequently considered for analysis.

#### 2.3.3. Repeated Sprinting Ability

The protocol applied to assess anaerobic performance was the “Running Anaerobic Sprint Test” (RAST) [48,49] through an infrared photocell system (Witty, Micro Gate System, Mahopac, NY, USA). The test consisted of 6 sprints of 35 m. Each sprint was performed with a 10 s active rest interval between them, during which players decelerated after crossing the 35 m timing gates, walked back to the starting line, and waited for the next start signal, with the pace of return self-regulated and the final 5 s counted down by the assessor. All RAST trials were conducted under similar environmental conditions, in the morning on a dry grass surface, with players wearing football boots. The RAST protocol has previously been shown to provide consistent and reproducible measurements in football players. Research demonstrates high reproducibility for Peak Power (ICC = 0.88, CV = 10.2%) and mean power (ICC = 0.96, CV = 5.9%) under field conditions using appropriate footwear, as well as on rigid surfaces [50]. These indices indicate minimal measurement error and robust test–retest reliability for RAST.

The following variables were obtained and calculated for analysis [48,49]: (1) total time of the six sprints (RAST TT); (2) Peak Power (RAST PP) = (Body Mass × Distance^2^) ÷ Time^3^; (3) Relative Peak Power (RAST RPP) = PP ÷ Body Mass; (4) Fatigue Index (RAST FI) = (maximum power − minimum power) ÷ total time of 6 sprints.

#### 2.3.4. Maximum Speed

The test comprised one trial of a 35 m linear sprint [51]. The effort time was obtained by the same device utilised in RAST, the infrared photocell system (Witty, Micro Gate System, Mahopac, NY, USA). Velocity was calculated by dividing distance by time.

#### 2.3.5. Change of Direction

**Figure 1:**
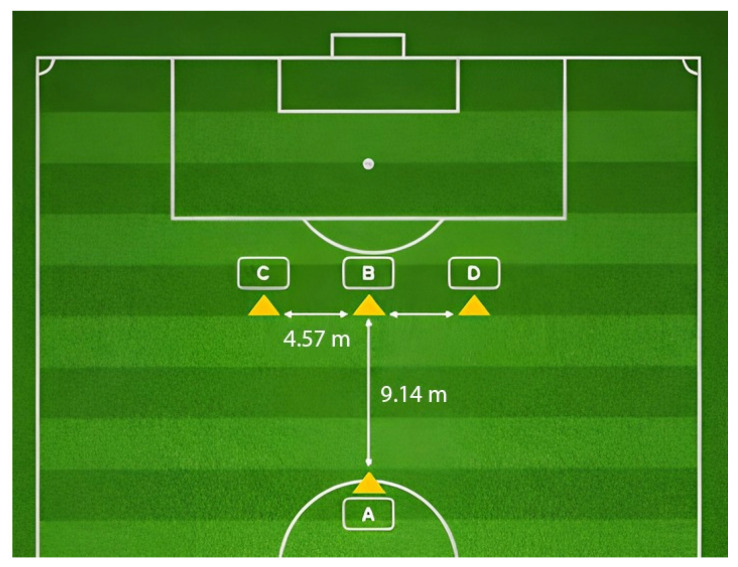
Visual Representation of the *t*-Test. The route starts at A to B ((1)—forward running), B to C ((2)—sideways running), C to D ((3)—sideways running), D to B ((4)—sideways running), and B to A ((5)—backward running).

Change of direction ability was assessed by the *t*-Test through the infrared photocell system (Witty, Micro Gate System, Mahopac, NY, USA), placed at the starting point, to measure the total time of the test [51,52]. The test consisted of 1 repetition along the route illustrated in Figure 1. The trial was not considered valid if at least one of the following criteria is not met: (a) the order of the route; (b) running sideways on courses (2), (3) and (4) (e.g., crossing the feet); and (c) not facing forwards throughout the whole test.

Although only a single repetition was performed in our protocol, previous research has demonstrated that the *t*-Test exhibits high reliability, with intraclass correlation coefficients (ICC) up to 0.95 and coefficients of variation (CV) around 1.96% [53].

#### 2.3.6. Aerobic Endurance

A continuous running test to exhaustion was applied to estimate cardiorespiratory endurance. Before the test, participants underwent anthropometric data collection (biographical information, weight, and height) to configure the testing software. The protocol used, known as the “Soccer Test,” followed standards described in the literature [54,55] and involved physical exertion with incremental maximal loads (speed and incline) on a motorised treadmill, using the K5 Wearable Metabolic Technology (K5WMT) device (Cosmed, Rome, Italy).

Before each assessment, the K5WMT underwent three mandatory calibrations: (1) calibration of the volume disturbance, (2) calibration of the sampling line (using the purifier), and (3) calibration of the benchmark gases. Then, the evaluators adjusted the torso and head harnesses to the athlete, connecting the equipment. A heart rate monitor was also placed around the diaphragm area (Garmin, HRM-Dual, Olathe, KS, USA). The test began with a warm-up at 8 km·h^−1^, increasing speed every 2 min until reaching 20 km·h^−1^. From 16 km·h^−1^ onward, a 2% incline was introduced, which gradually increased by 2% at each subsequent stage. The test protocol continued until volitional exhaustion (athlete’s request to stop), with mean test durations typically ranging from 12 to 15 min (including the initial 2 min warm-up at 8 km·h^−1^), consistent with the observed values in this study (SG: 15.54 ± 3.12 min; YG: 14.44 ± 2.41 min). Maximal effort was confirmed by the subject’s exhaustion, followed by a 2 min recovery phase at 4 km·h^−1^ (not included in total test time), in accordance with similar protocols used for soccer players [54,55]. After the test, the equipment was removed, and the results, such as VO2max and total test time (K5 TT), were immediately provided by the software and used for analysis.

### 2.4. Statistical Analysis

Statistical analysis was conducted using SPSS version 28.0 (SPSS et al. Company, Armonk, NY, USA). Variables analysed were expressed by groups (SG and YG). Before conducting statistical analysis, the normality of the research variables was assessed using the Shapiro–Wilk test. Statistical significance was set at 5%.

Given that not all datasets had a normal distribution, the Mann–Whitney U test was employed to compare the distributions of the different assessments between the SG and YG. Effect sizes (r) for the Mann–Whitney U test were calculated in Microsoft Excel for Microsoft 365 (version 2510, 64-bit) by using the formula $r=\frac{Z}{\sqrt{N}}$, where Z is the standardised test statistic, and N is the total sample size. The results were interpreted according to the following Cohen’s thresholds [56]: |r| < 0.1 (trivial), 0.1 ≤ |r| < 0.3 (small), 0.3 ≤ |r| < 0.5 (medium), and |r| ≥ 0.5 (large).

Spearman (for variables without normal distribution) and Pearson (for variables with normal distribution) correlations were conducted between JH and the remaining performance variables. Throughout this correlation analysis, Hopkins’ interpretive guidelines (https://sportsci.org/ (accessed on 22 December 2025)) were adopted to assess the strength of the correlation coefficients: trivial (0–0.1), small (0.1–0.3), moderate (0.3–0.5), large (0.5–0.7), or very large (0.7–0.9) [28,29].

Simple linear regression analyses were performed separately within each group to evaluate the predictive capacity of JH on the remaining physical performance outcomes. Cohen’s f^2^ was calculated as an effect size measure for regression models, interpreted according to Cohen’s conventional thresholds: f^2^ ≥ 0.02 (small), f^2^ ≥ 0.15 (medium), f^2^ ≥ 0.35 (large) [57,58]. Linear regression assumptions (normality, homoscedasticity, independence) were checked using residual histograms, Normal P–P plots, ZPRED vs ZRESID scatterplots and Durbin–Watson statistics (1.62–2.46). Diagnostic tests for multicollinearity, including the variance inflation factor (VIF = 1.000) and tolerance (1.000), confirmed the absence of collinearity issues. No violations were observed. Only significant models were presented.

The neuromuscular profile of the players was analysed using parametric tests based on the normal distribution of JH across the overall sample. Comparisons between groups were performed using independent samples *t*-tests for JH, with effect sizes calculated using Cohen’s d. Differences between playing positions within the same group were examined using one-way ANOVA. Cohen’s d-values were interpreted according to the thresholds proposed by Hopkins et al. (2009) [59]: d ˂ 0.2 (trivial), 0.2 ≤ d < 0.6 (small), 0.6 ≤ d < 1.2 (moderate), 1.2 ≤ d < 2.0 (large), 2.0 ≤ d < 4.0 (very large) and d ≥ 4.0 (nearly perfect).

Comparisons of identical positions between teams were performed using independent samples *t*-tests for JH (with Cohen’s d effect sizes). Positional comparisons were treated as exploratory analyses, given the limited statistical power.

## 3. Results

### 3.1. Group Results

Table 3 below presents the mean results for the assessed parameters. It is shown that the SG had better performances in all the variables, except for the RAST FI (SG = 0.39 ± 0.14 vs. YG = 0.33 ± 0.08) and the K5 Max VO2 (SG = 53.74 ± 6.49 mL·kg^−1^·min^−1^ vs. YG = 56.68 ± 5.55 mL·kg^−1^·min^−1^).

When comparing groups, the distribution of scores for the various metrics studied showed significant differences. Specifically, SG obtained significantly greater results concerning JH, Abduction Strength DL, Abduction Strength NDL, and RAST PP. On the other hand, the YG had significantly better values for RAST FI and K5 Max VO2, as illustrated in Table 4 below.

### 3.2. Jump Height: Relative Importance and Predictive Value

#### 3.2.1. Correlations

In the SG, our results show moderate significant correlations between RAST RPP and JH (R = 0.43, *p* = 0.012), as well as between Maximum Speed and JH (R = 0.39, *p* = 0.022). Regarding the YG, we only verified a significant moderate correlation between K5 TT and JH (R = 0.34, *p* = 0.035).

#### 3.2.2. Linear Regressions

Simple regression analyses (Table 5) conducted for the SG indicated that JH was a significant predictor for several key performance variables. Specifically, JH significantly predicted Maximum Speed (R^2^ = 0.239, *p* = 0.003, f^2^ = 0.314), RAST PP (R^2^ = 0.291, *p* = 0.001, f^2^ = 0.410), and RAST RPP (R^2^ = 0.467, *p* < 0.001, f^2^ = 0.876), ranging from medium to large effect sizes. In all models, higher JH values were associated with superior performance outcomes, as indicated by positive standardised coefficients and confidence intervals that did not cross zero. For the YG, JH significantly predicted K5 Total Time (R^2^ = 0.114, *p* = 0.035, f^2^ = 0.129), with a small effect size, demonstrating a modest explanatory value. No other models in the YG achieved statistical significance.

### 3.3. Neuromuscular Power Profile

Table 6 below illustrates the mean values obtained according to playing position in each group.

#### 3.3.1. Group Comparisons

The results show that the SG had significantly higher values of JH, with moderate effect sizes (*p* = 0.002, 95% CI: [1.350; 5.561]; Cohen’s d = 0.753, 95% CI: [0.283; 1.217]), representing a 9.5% greater mean JH compared to the YG.

#### 3.3.2. Within-Group Positional Comparisons

No significant differences in JH were found across the various playing positions within each group.

#### 3.3.3. Between-Group Positional Comparisons

When examining positional comparisons between groups, SG Defenders demonstrated a significantly greater mean JH than YG Defenders, with a difference of approximately 12.4% (*p* = 0.021, 95% CI: [0.738; 8.447]; Cohen’s d = 0.935, 95% CI: [0.137; 1.717]). Similarly, SG Midfielders outperformed YG Midfielders, jumping on average 12.2% higher (*p* < 0.001, 95% CI: [2.020; 6.508]; Cohen’s d = 1.690, 95% CI: [0.690; 2.659]), representing moderate (for DF) and large (for MF) effect sizes, respectively.

While no statistically significant differences were observed for the other positions, SG Forwards nevertheless jumped 7.6% higher than those in the YG. In contrast, the trend reversed for Goalkeepers, with SG Goalkeepers jumping approximately 9.8% less than their YG counterparts.

## 4. Discussion

This study demonstrates that senior soccer players exhibited better athletic performance than youth athletes, a finding consistent with established knowledge regarding the progressive improvement of physical performance across competitive levels. The SG presented superior results in almost every physical variable analysed, particularly for JH, abduction strength (DL and NDL), and RAST PP. These outcomes align with previous studies [12,13,14,15,16] that report fitness levels, neuromuscular capacity, and overall physical profiles become more developed as players progress from youth to senior categories. Such improvements are likely explained by the greater physical demands, training exposure, and competitive intensity characteristic of professional environments [19,60].

This pattern is also supported by recent studies [61,62], which indicate that exposure to neuromuscular and integrative training programmes in youth is associated with substantial gains in JH, explosive power, and overall physical fitness. Although this cannot be confirmed by the present cross-sectional design, these adaptations may be related to maturational processes in neural activation, motor unit recruitment, and improved coordination, fundamental factors for translating training stimuli into enhanced performance as athletes age. Notably, the responsiveness of JH to targeted training in youth and its predictive value in seniors might reflect how neuromuscular qualities mature over time and could potentially be optimised by structured exposure [61,62]. Furthermore, Loturco et al. (2020) [63] highlighted that change of direction, speed, and jump performance tend to improve with age and competitive level, reflecting the sensitivity of these capacities as markers of athletic maturation and readiness. Moreover, another study [60] also found that professional soccer players outperformed younger categories in strength and power (assessed through squat and CMJ), underlining the need for tailored training approaches to address developmental demands, particularly in youth athletes.

Notably, the YG achieved better results than the SG in the RAST FI (SG = 0.39 ± 0.14 vs. YG = 0.33 ± 0.08) and Max VO2 (SG = 53.74 ± 6.49 mL·kg^−1^·min^−1^ vs. YG = 56.68 ± 5.55 mL·kg^−1^·min^−1^), consistent with previous evidence demonstrating superior aerobic capacity in youth soccer players compared to seniors [64]. These findings suggest that younger athletes may maintain stronger aerobic endurance and recovery capacities, which could be related to reduced exposure to the off-season detraining effects or a more sustained conditioning regimen emphasising base endurance.

Regarding the relative importance and predictive value of JH, this metric emerged as a meaningful predictor of athletic potential, particularly as players progress towards elite and senior levels. Specifically, in the SG, JH showed moderate significant associations with anaerobic power and sprint performance (RAST RPP and JH, R = 0.43, *p* = 0.012; Maximum Speed and JH, R = 0.39, *p* = 0.022). In the SG, JH significantly predicted RAST PP (R^2^ = 0.291), RAST RPP (R^2^ = 0.467), and maximum speed (R^2^ = 0.239), with medium to large effect sizes (f^2^ = 0.314–0.876), suggesting that JH may be an important indicator of high-level physical performance. In the YG, we identified a single significant moderate correlation between K5 TT and JH (R = 0.34, *p* = 0.035), with JH only modestly predicting K5 TT (R^2^ = 0.114, *p* = 0.035), indicating a small effect size (f^2^ = 0.129). In all models, higher JH values corresponded to superior performance outcomes. These findings are consistent with meta-analytic evidence suggesting that JH is a highly sensitive marker of neuromuscular maturation and training adaptation in young athletes, particularly when integrative neuromuscular training is prioritised over traditional methods [62].

This pattern may reflect youth training priorities that emphasise skill acquisition, technical–tactical development, and game intelligence rather than targeted anaerobic power conditioning, although this remains a hypothesis that cannot be directly tested with the current cross-sectional data. The emphasis on higher volumes of these field-based activities could indirectly sustain aerobic base development while potentially limiting the maturation of anaerobic capacities. Consequently, JH–anaerobic power relationships in youth players may be weaker when training practices focus mainly on skill acquisition and technical fundamentals instead of competitive outcomes [65]. This may suggest that the role of JH in predicting physical performance matures as athletes develop, likely reflecting a shift towards neuromuscular dominance at higher competitive standards. In practical terms, profiling athletes using JH provides coaches with a robust tool for monitoring developmental readiness, guiding talent identification, and tailoring individualised training strategies from youth stages into elite, senior competition [62].

Overall, these results indicate that JH, while more predominant and predictive at the senior level, likely reflects a neuromuscular quality that develops with maturation and is characteristic of elite-level readiness. This reinforces its relevance for selection and programming at advanced stages and corroborates the existing literature showing that higher JH is generally associated with better physical performance [2,30]. Consequently, this might underscore the potential value of integrating plyometric and power-based training interventions early in youth development programmes [66]. The literature thus supports the early application of plyometric and multi-component neuromuscular programmes in youth, indicating that such strategies are associated with faster jump-related adaptations and help to bridge physical gaps between groups, while offering lasting benefits in performance progression and injury resilience across athletic careers [62]. Furthermore, Hammami et al. (2025) [61] emphasise that improvements in neuromuscular parameters such as JH during adolescence are also accompanied by positive shifts in psychological well-being and self-confidence, attributes closely linked to the ability to meet the demanding transitions between youth and senior football. These developmental correlations support the potential utility of JH not only as an indicator for identifying youth athletes with above-average physical potential but also as a broader marker of competitive preparedness, supporting talent identification and the strategic allocation of developmental resources within soccer academies.

Additionally, a similar phenomenon has already been observed in different, and much faster, athletic populations (e.g., elite sprinters and jumpers) who exhibited strong and significant correlations across a wide range of sprint distances (10 to 60 m) and heights achieved in various jump tests (e.g., loaded and unloaded squat and CMJ, as well as drop jumps from 45 and 75 cm) [67]. Therefore, it appears that JH can serve as a valid predictor of sport performance across different disciplines, corroborating the findings presented here.

When analysing jump performance by group and playing position, mean JH values (SG = 39.95 ± 4.74 cm; YG = 36.49 ± 4.46 cm) corresponded well with normative data in the literature [2], which reports CMJ’s JH ranging from 33.6 to 57.2 cm in senior players and 34.8 to 58.6 cm in elite youth players. However, the significant group differences in JH (seniors registering a 9.5% greater group average, *p* = 0.002, with moderate effect sizes) contrast with some previous findings suggesting that CMJ performance does not necessarily differ between elite junior and professional levels [68] and that vertical jump improvements during maturation may stabilise after the U15 category [63]. These discrepancies probably reflect methodological differences, training regimens, or sample-specific characteristics, reinforcing the need for continued longitudinal tracking of neuromuscular profiles in player development.

Concerning positional comparisons, previous research [69,70,71,72,73,74] has investigated maximal JH among different playing positions in soccer, yielding mixed results. Hassen et al. (2023) [74] state that some studies found no significant differences in jump performance based on position, whereas others reported disparities in jumping between outfield players and GK and across different outfield positions. In contrast, no major disparities were observed in our results. These JH findings may be due to the small number of players per position or indicative of a balanced development capacity of this parameter within each group.

Regarding between-group positional comparisons, we observed that senior DF and MF tended to jump higher than their youth peers (by approximately 12.4% and 12.2%, respectively), although these positional differences should be viewed as exploratory given the limited sample sizes per role. This suggests that jump performance gains may accumulate with age and exposure, particularly in roles that demand repeated explosive actions. FW in the SG also showed a trend towards higher jump ability, though without statistical significance. A particularly intriguing result was observed for GK, where youth players appeared to outperform their senior counterparts by approximately 9.8% in JH. This reversed pattern may be partly attributable to the small sample size in our study (N = 2 SG vs. N = 7 YG) and cohort-specific characteristics. Given the critically small samples, these findings should be considered preliminary and interpreted with caution. The exploratory nature and reduced statistical power of these comparisons highlight the need for further investigation with larger and more representative groups.

This study provides novel data that objectively characterise JH as the key jump performance metric influencing other athletic parameters among youth and senior soccer players from the same club. Additionally, it evaluates the jumping profile of this population according to age category and playing position. These findings are crucial for guiding successful transitions from youth to senior and elite levels, for monitoring and addressing the specific demands of each category and position, as well as for supporting decision-making in player recruitment and development strategies.

Nevertheless, there are some limitations inherent to this research. First, the subdivided sample size across playing positions and age groups was relatively small, especially in specific roles such as GK, which substantially constrains statistical power and broader generalisability of our findings. As a result, position-specific inferences should be clearly regarded as exploratory and preliminary. Nonetheless, the literature includes examples of similarly small samples in position-specific comparisons [3,10]. Second, the heterogeneity within the SG, stemming from the combination of professional athletes from both the 2nd and 4th Portuguese divisions, may introduce confounding factors. Although both subgroups followed comparable professional routines and training loads within the same club structure, this mixed competitive level means that the neuromuscular profile of the SG should be interpreted as reflecting a blended professional context rather than a homogeneous top-division cohort. The generalisability of these results to other senior populations is therefore limited. Additionally, all participants came from the same club and training system, which limits the external validity and generalisation of results to broader sporting contexts. Furthermore, the absence of randomisation in test order and limited control over fatigue may affect internal validity. Lastly, the cross-sectional nature of our study precludes direct causal inferences regarding the development of JH over time and its relationship to maturation, training exposure, and performance. Additionally, it is worth noting that the study sample consisted solely of male athletes, and data regarding their biological maturation stage, prior experience, and injury history were not available. These factors may influence neuromuscular performance and physical test outcomes and should be considered when interpreting the findings. Future research would benefit from including these variables to provide a more comprehensive understanding of their impact on athletic development and performance.

Furthermore, considering the present outcomes, future studies should investigate the association between neuromuscular measures and external performance variables monitored via Global Positioning System (GPS) in order to reinforce the practical applicability of these indicators. Firstly, it would be relevant to examine whether the JH can predict the capacity to perform high-intensity efforts during competition, specifically sprint frequency and high-speed running distance, thereby clarifying its value as a predictor of match performance. Secondly, research should investigate the relationship between acute reductions in JH in pre- and post-match contexts and subsequent changes in high-intensity outputs recorded by GPS, with the aim of validating jump tests as tools for monitoring fatigue and competitive readiness. Finally, it is suggested that future investigations assess whether players with higher JH demonstrate greater ability to sustain repeated high-intensity efforts, particularly under conditions of congested match schedules, which could provide valuable insights for rotation strategies, individualised training, and the prevention of accumulated fatigue.

## 5. Conclusions

This study suggests that JH is a reliable and practical indicator of neuromuscular performance and physical readiness in soccer players across various age groups and playing positions. As athletes progress to higher competitive levels, JH becomes a stronger predictor of performance, showing large effects and robust associations with anaerobic and maximal-effort metrics such as relative Peak Power and sprint speed. For youth players, JH remains meaningfully linked to aerobic endurance, highlighting its group-specific relevance, although causality cannot be inferred from the cross-sectional and correlational nature of our analysis.

Group and positional analyses revealed significantly greater JH in senior DF and MF compared to their youth peers, suggesting that accumulated gains occur with age and training exposure. Differences between matched positions across groups further support the notion that JH distinguishes between advanced neuromuscular capacity and competitive maturity. However, findings are strictly exploratory due to the limited sample size. The single-club context also constrains generalisability to broader populations.

Practitioners should therefore consider prioritising JH for profiling advanced players. Overall, these findings support the use of routine jump height assessments as a core tool for performance profiling and player monitoring in soccer. The simplicity, practicality, and standardised nature of jump-based evaluations make them valuable for assessing physical status, guiding individualised training, and informing talent identification and recruitment.

## Figures and Tables

**Table 1:** Sample distribution by group, playing position, age, and anthropometric descriptive statistics.

Group	Position	N	Age (Years), Mean ± SD	Height (cm), Mean ± SD	Body Mass (kg), Mean ± SD
**SG**	GK	2			
	DF	16			
	MF	11			
	FW	7			
	Total	36	23.72 ± 4.60	180.94 ± 6.72	77.71 ± 7.95
**YG**	GK	7			
	DF	13			
	MF	12			
	FW	12			
	Total	44	17.20 ± 0.73	178.43 ± 6.23	70.03 ± 6.09

**SG**, Senior Group; **YG**, Youth Group; **GK**, Goalkeepers; **DF**, Defenders; **MF**, Midfielders; **FW**, Forwards. Note: Age, height, and body mass are reported at group level only to avoid overly small subgroups and minimise the risk of indirect identification of individual players.

**Table 2:** Number of cases by test variable.

Variables	SG (N)	YG (N)
CMJ JH, cm	35	41
Adduction Strength DL, kg·f	33	35
Adduction Strength NDL, kg·f	33	35
Abduction Strength DL, kg·f	33	35
Abduction Strength NDL, kg·f	32	35
Maximum Speed, km·h^−1^	34	42
*t*-Test—COD, s	32	42
RAST TT, s	33	42
RAST PP, W	33	42
RAST FI	33	42
RAST RPP, W·kg^−1^	33	42
K5 Max VO2, mL·kg^−1^·min^−1^	36	41
K5 TT, min	35	41

**SG,** Senior Group; **YG,** Youth Group; **JH**, Jump Height; **DL**, Dominant Leg; **NDL**, Non-Dominant Leg; **COD**, Change of Direction; **TT**, Total Time; **PP**, Peak Power; **FI**, Fatigue Index; **RPP**, Relative Peak Power; **Max VO2**, Maximum Oxygen Uptake.

**Table 3:** Mean, standard deviation, and sample size for the variables in the study.

Variables	Groups			
	SG		YG	
	Mean	Std. Dv.	Mean	Std. Dv.
CMJ JH, cm	39.95	4.74	36.49	4.46
Adduction Strength DL, kg·f	48.15	7.09	47.71	7.64
Adduction Strength NDL, kg·f	48.61	6.66	48.58	8.21
Abduction Strength DL, kg·f	49.70	6.68	42.97	6.60
Abduction Strength NDL, kg·f	51.01	7.72	43.58	6.53
Maximum Speed, km·h^−1^	26.18	1.23	25.96	1.14
*t*-Test—COD, s	9.16	0.38	9.17	0.32
RAST TT, s	31.84	1.45	31.93	1.19
RAST PP, W	853.95	184.30	716.99	110.77
RAST FI	0.39	0.14	0.33	0.08
RAST RPP, W·kg^−1^	10.94	1.94	10.20	1.20
K5 Max VO2, mL·kg^−1^·min^−1^	53.74	6.49	56.68	5.55
K5 TT, min	15.54	3.12	14.44	2.41

**SG,** Senior Group; **YG,** Youth Group; **JH**, Jump Height; **DL**, Dominant Leg; **NDL**, Non-Dominant Leg; **COD**, Change of Direction; **TT**, Total Time; **PP**, Peak Power; **FI**, Fatigue Index; **RPP,** Relative Peak Power; **Max VO2**, Maximum Oxygen Uptake.

**Table 4:** Comparison of the different assessments between the SG and YG.

Mann–Whitney U Test			
	Z	Asymp. Sig. (2-Tailed)	Effect Size (r)
CMJ JH, cm	−3.314	<0.001	0.371 (medium)
Adduction Strength DL, kg·f	−0.534	0.593	0.060 (trivial)
Adduction Strength NDL, kg·f	−0.374	0.708	0.042 (trivial)
Abduction Strength DL, kg·f	−3.713	<0.001	0.415 (medium)
Abduction Strength NDL, kg·f	−3.754	<0.001	0.420 (medium)
Maximum Speed, km·h^−1^	−0.611	0.541	0.068 (trivial)
*t*-Test—COD, s	−0.464	0.643	0.052 (trivial)
RAST TT, s	−0.576	0.564	0.064 (trivial)
RAST PP, W	−3.416	<0.001	0.382 (medium)
RAST FI	−2.658	0.008	0.297 (small to medium)
RAST RPP, W·kg^−1^	−1.681	0.093	0.188 (small)
K5 Max VO2, mL·kg^−1^·min^−1^	−2.083	0.037	0.233 (small)
K5 TT, min	−1.554	0.120	0.174 (small)

**SG,** Senior Group; **YG**, Youth Group; **JH**, Jump Height; **DL**, Dominant Leg; **NDL**, Non-Dominant Leg; **COD**, Change of Direction; **TT**, Total Time; **PP**, Peak Power; **FI**, Fatigue Index; **RPP**, Relative Peak Power; **Max VO2**, Maximum Oxygen Uptake.

**Table 5:** Simple regression analyses results for both groups. Only significant models are presented.

JH									
Group	Variables	R^2^	Adjusted R^2^	Cohen’s f^2^ (Effect Size)	Model Fit ANOVA Sig.	Unstandardised Coefficient (β)	Standardised Coefficient (β)	Sig.	95% CI
**SG**	Maximum Speed	0.239	0.215	0.314	0.003 *	0.130	0.489	0.003 *	[0.047; 0.214]
	RAST PP	0.291	0.268	0.410	0.001 *	21.867	0.540	0.001 *	[9.372; 34.363]
	RAST RPP	0.467	0.450	0.876	<0.001 *	0.292	0.684	<0.001 *	[0.178; 0.407]
**YG**	K5 TT	0.114	0.090	0.129	0.035 *	0.184	0.338	0.035 *	[0.013; 0.354]

* Significant *p*-value. **JH**, CMJ Jump Height; PP, Peak Power; **RPP**, Relative Peak Power; **TT**, Total Time.

**Table 6:** Groups’ neuromuscular power profile by playing position (SG and YG).

Position	Senior Group	CMJ Jump Height (JH), cm	Youth Group	CMJ Jump Height (JH), cm
**Goalkeeper (GK)**	N	2	N	7
	Mean	35.85	Mean	39.76
	Std. Deviation	10.82	Std. Deviation	5.73
**Defender (DF)**	N	16	N	12
	Mean	41.65*	Mean	37.06
	Std. Deviation	5.09	Std. Deviation	4.65
**Midfielder (MF)**	N	11	N	11
	Mean	39.34 *	Mean	35.07
	Std. Deviation	1.94	Std. Deviation	2.99
**Forward (FW)**	N	6	N	11
	Mean	37.88	Mean	35.21
	Std. Deviation	4.72	Std. Deviation	3.89
**Total**	N	35	N	41
	Mean	39.95 *	Mean	36.49
	Std. Deviation	4.74	Std. Deviation	4.46

* Significant difference between SG and YG for the corresponding row (DF, MF, and overall group mean; *p* < 0.05). **Note:** Positional comparisons are exploratory.

## Data Availability

Data will be provided to all interested parties upon reasonable request. The data are not publicly available due to privacy and ethical restrictions.
